# Addiction to Cosmetic Procedures: A Scoping Literature Review

**DOI:** 10.1007/s00266-025-05153-8

**Published:** 2025-08-12

**Authors:** Wendy Wagenaar, Anne-Mette Hermans

**Affiliations:** Tranzo, Tilburg School of Social and Behavioral Sciences, Tilburg University, Professor Cobbenhagenlaan 125, 5037 DB Tilburg, The Netherlands

**Keywords:** Cosmetic procedures, Addiction, Scoping review, Excessive use, Psychological dependence, Behavioral addiction

## Abstract

Over the past few decades, cosmetic procedures have become increasingly popular. Alongside this rise in popularity, however, concerns have been raised regarding cosmetic extremes and potential “addiction” to cosmetic procedures. Yet, it remains unclear and underspecified what this “addiction” entails and/or how it can be established. This scoping review aims to (1) compile and explore articles defining “addiction” to cosmetic procedures and (2) identify key characteristics which in the literature are related to the concept of “addiction” to cosmetic procedures. In accordance with the JBI methodology, we conducted a scoping review, searching PubMed, Web of Science and PsycINFO for relevant articles published between January 2000 and January 2024. The eligibility criteria included all English-language articles that described characteristics associated with “addiction” to cosmetic procedures. Ultimately, 13 studies met the inclusion criteria, highlighting the scarcity of research on this topic. The reviewed studies used a variety of terms to describe “addiction” to cosmetic procedures. In addition, the studies indicated characteristics that are related to the concept of “addiction” to cosmetic procedures, such as “excessive” use; preoccupation with cosmetic procedures; hazardous use; and psychological dependence. This review represents a critical step in exploring the concept of “addiction” to cosmetic procedures. Based on this review, it would be desirable to further conceptualize and investigate whether “addiction” to cosmetic procedures could and/or should be recognized as a behavioral addiction.

*Level of Evidence V* This journal requires that authors assign a level of evidence to each article. For a full description of these Evidence-Based Medicine ratings, please refer to the Table of Contents or the online Instructions to Authors www.springer.com/00266.

**Supplementary Information:**

The online version contains supplementary material available at 10.1007/s00266-025-05153-8.

## Introduction

Over the past couple of decades, cosmetic procedures have become increasingly popular. In 2023, 35 million cosmetic procedures were performed worldwide, including both surgical procedures, such as breast augmentations and rhinoplasties, and non-surgical procedures, such as botulinum toxin injections, filler and laser treatments and chemical peels, among others [1, 2]. As the number of cosmetic procedures rises, there is growing concern about some of the unrealistic expectations people have regarding these procedures [3]. One of these concerns relates to the (hypothesized) group of people who have unrealistic expectations and undergo many (“excessive”) procedures. This demand for “excessive” procedures is sometimes described as an “addiction”^1^ to cosmetic procedures. However, it is unclear what this addiction to cosmetic procedures entails and/or how it can be established.

Although various studies have emphasized the low complication rate after cosmetic procedures [4, 5], there are still risks associated with these procedures [6], such as permanent or semi-permanent physical disfigurement or loss of function [7–9]. This is even more concerning when individuals become “addicted” to cosmetic procedures, increasing the risk of adverse reactions. For example, two case studies of patients showed significant complications due to their “excessive” demand for cosmetic injections [10, 11]. These potential complications underscore the need to address the concept of addiction to cosmetic procedures.

Although the phenomenon of addiction to cosmetic procedures has not been formally defined in the literature, anecdotal stories from popular media indicate the potential existence of patients with such addictive behaviors [12, 13]. Traditionally, addiction has been associated with substance abuse or dependence, but there is growing recognition of behavioral addictions, including (but not limited to) gambling [14], internet use [15], video-game playing [16], sex [17], exercising [18], food [19] and shopping [20]. A behavioral addiction is similar to drug addiction in that the individual becomes addicted to the behavior or the feeling it produces, however, unlike with drug addiction, without the dependence on a particular substance. Despite the absence of physical signs typical of drug addiction, behavioral addictions share clinical expressions like cravings, tolerance and withdrawal symptoms, as well as comorbidity, neurobiological profiles, heritability and treatment options [21, 22]. Precursors to behavioral addiction can include psychopathologies such as depression, substance dependence, withdrawal, social anxiety and lack of social support [23]. These behavioral addictions are considered in the “Substance-Related and Addictive Disorders” section of DSM-5, where gambling disorder is the only behavioral addiction included as a diagnosable condition [21]. Other addictive behaviors, such as internet gaming and shopping, were considered for inclusion, but the data for these types of behavioral addictions are currently too limited [24]. While the concept of addiction to cosmetic procedures may share characteristics and precursors with other behavioral addictions, it remains unclear to what extent these similarities exist.

Consequently, an important step toward addressing this issue is a broad exploration of its conceptualization in the field. To our knowledge, no comprehensive review on the conceptualization of addiction to cosmetic procedures has been established to date. Therefore, the aim of the present scoping review was to (1) compile and explore articles defining addiction to cosmetic procedures; and (2) identify key characteristics and precursors which in the literature are related to the concept of addiction to cosmetic procedures.

## Methods

This scoping review was conducted in accordance with the JBI methodology for scoping reviews [25]. The objective was to compile and analyze articles conceptualizing addiction to cosmetic procedures [26]. The primary search was conducted using PubMed. The search string was carefully developed by incorporating terms related to addiction, which were drawn from existing reviews on behavioral addiction [27, 28]. Similarly, the search terms related to cosmetic procedures were based on previous reviews conducted in the field [29, 30]. The search strategy was refined through consultations with a specialized university librarian, resulting in the following search string:

(“cosmetic techniques”[mesh] AND “compulsive behavior”[mesh]) OR (“cosmetic techniques”[mesh] AND “behavior, addictive”[mesh]) OR ((“cosmetic*”[tiab] OR “esthetic*”[tiab] OR “aesthetic*”[tiab] OR “plastic surger*”[tiab] OR “botulinum toxin”[tiab] OR “dermal filler”[tiab]) AND (“addict*”[tiab] OR “compuls*”[tiab] OR “obsess*”[tiab] OR “disorder”[tiab] OR “patholog*”[tiab]))

To expand the scope of the search, additional searches were conducted using Web of Science and PsycINFO. The terms used for these additional searches were the same as those used in the initial PubMed search and tailored to the specific search engine. Articles were selected through a hierarchical screening process conducted by two researchers using the Rayyan Qatar Computing Research Institute software (https://www.rayyan.ai). After duplicates were removed, studies were selected according to the following inclusion criteria: They (1) describe “addiction” to cosmetic procedures; (2) are published in the period between January 2000 until the 1 st of February 2024; (3) provide a full-text article; (4) are published in English. Studies that focused exclusively on body dysmorphic disorder (BDD) without addressing “addictive tendencies” were excluded to ensure the review specifically included papers on addictive behaviors. Disagreements in article selection were resolved through discussion.

Key data related to the definition and characteristics of the concept of addiction to cosmetic procedures were systematically extracted. To analyze the extracted articles, we conducted a directed content analysis. A directed content analysis allows for the application of existing theories or frameworks while also enabling new evidence to emerge through open coding. For this analysis, a coding guide was developed, based on key characteristics identified by previous reviews on cosmetic procedures [29, 30] and existing frameworks for behavioral addictions [27, 31, 32]. Additionally, open coding was employed to identify additional categories and subcategories. Following the initial coding process, the categories, subcategories, codes and examples were reviewed and refined, with the guide being developed iteratively over multiple stages.

## Results

**Fig. 1:**
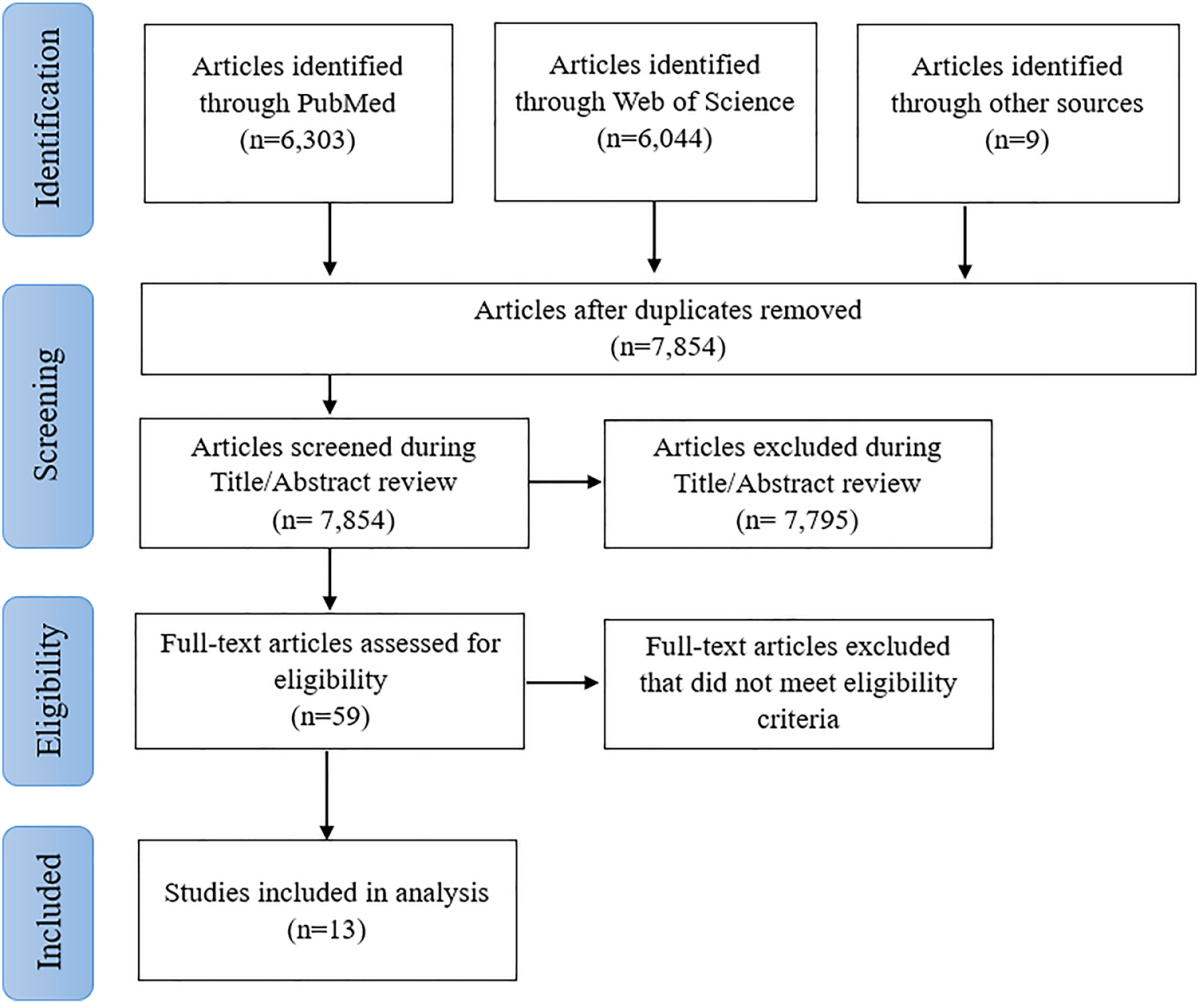
Diagram of study selection processes

The initial search yielded 12,356 references. After discarding duplicates, 4502 articles were excluded, leaving 7854 articles for Title/Abstract screening. Subsequently, 59 of these articles were selected for a comprehensive full-text review. Ultimately, 13 articles met our inclusion criteria and were included in the final review. The complete flowchart detailing the study selection process in accordance with the JBI methodology for scoping reviews is summarized in Fig. 1.

The studies included in the analysis encompassed a variety of methodologies. Specifically, our review covered five empirical reviews, three case reports, three cross-sectional studies, a single qualitative study and a single editorial. In terms of nomenclature, the reviewed studies employed a variety of terms for addiction to cosmetic procedures, including “cosmetic addiction” (*n* = 1), “cosmetic surgery addiction” (*n* = 1), “addiction to plastic surgery” (*n* = 1), “filler-addicted” (*n* = 2), “polysurgery addiction” (*n* = 2) and “surgery junkies” (*n* = 1) among others. The characteristics of the studies, including the terms and definitions used, are summarized in Table 1.

**Table 1:** Terms and definitions used in included articles

Author	Year	Study type	Term in text	Definition in text
Harth and Hermes [40]	2007	Empirical review	“Polysurgical addiction”	“Often a liking of or frenzy for surgery exists (...) and can particularly be observed in elective cosmetic surgery”
Suissa [37]	2008	Empirical review	“Addiction to plastic surgery”	“A disorder affecting the psychosocial image of the self”
Gimlin [46]	2010	Qualitative study	“Surgery junkies,” “surgical others,” “surgery addicts”	“Women, whether real or imagined, whose relationship with cosmetic procedures is at best, problematic and at worst, pathological”
Morioka and Ohkubo [43]	2014	Empirical review	“Polysurgery addicts”	“They were dissatisfied with the results of their previous surgery, disagreed with our suggestions, and attempted multiple procedures, but they were not persistent about one body part and frequently changed the part with which they were preoccupied.”
Rongioletti et al. [10]	2015	Case report	“Filler-addicted”	“Having had multiple procedures of filler injections into their face in the previous years at different times”
Lin et al. [11]	2017	Case report	“Addicted to cosmetic fillers”	“Patients interested in achieving a younger appearance may receive multiple filler injections, and they are often reluctant to disclose previous treatment, which leads to difficulty in making a diagnosis when complications occur”
D’Agostino et al. [38]	2018	Cross-sectional study	“Compelling use of cosmetic surgery”	“The compulsive way in which many people approach these procedures”
Ip and Ho [35]	2019	Case report	“Cosmetic ‘addiction’”	“People demonstrating a compulsive need for surgical interventions”
Jafferany et al. [36]	2020	Empirical review	−	“Seeking various cosmetics procedures present to aesthetic surgery clinics and demanding procedures which are out of proportion”
Sarwer [42]	2021	Editorial	“Obsessive-compulsive disorder” “in the case of aesthetic procedures”	“Where the compulsive behaviors are preceded by obsessions, defined as recurrent, persistent, and unwanted thoughts, urges, or impulse”
Shah et al. [45]	2021	Cross-sectional study	“Addictive like behaviors”	“Patients (...) who meet initial exploratory criteria for an SRD for cosmetic intervention”
Kim [41]	2022	Cross-sectional study	“Cosmetic surgery addiction”	“An obsession to repeatedly modify one’s appearance through cosmetic surgery”
Macovei et al. [39]	2023	Systematic review	“Bleachorexia,” “tooth whitening addiction”	“People who engage in excessive and compulsive behaviours related to teeth whitening procedures"

Based on a directed content analysis of the included studies [33, 34], Table 2 summarizes the key characteristics of addiction to cosmetic procedures, along with representative examples from the content coded. The reviewed studies employed various characteristics to define addiction to cosmetic procedures, as presented in Table 2. The characteristic of “repetitive and excessive use” emerged as the most prevalent, which includes “multiple and repeated procedures” (*n* = 10) and “excessive and out of proportion” use of cosmetic procedures (*n* = 6). For instance, studies described patterns of patients repeatedly seeking surgeries [35, 36], visiting several cosmetic surgeons or dermatologists [11] and continuing to be obsessed with altering other body parts [37].

**Table 2:** Directed content analysis of the key characteristics related to addiction to cosmetic procedures

Category	Sub-category	Examples
Repetitive and excessive use	Multiple and Repeated Procedures (*n* = 10)	“Multiple, sequential cosmetic injections,” “visit several cosmetic surgeons or dermatologists,” “other body parts that must be changed and transformed”
	Excessive and Out of Proportion (*n* = 6)	“Excessive demand of multiple cosmetic injections,” “demanding procedures which are out of proportion”
Preoccupation with behavior	Obsessive (*n* = 6)	“Obsession to repeatedly modify one’s appearance through cosmetic surgery,” “obsessive demand”
	Compulsive (*n* = 4)	“Compulsive way which many people approach these procedures,” “compulsive need for surgical interventions”
Hazardous use	Denial of Potential Risks (*n* = 5)	“Push aside possible risks,” “without taking in consideration all risks and possible adverse effects”
	Increasing Adverse Outcomes (*n* = 2)	“Increasing the risk of adverse reactions,” “she also suffered from serious side effects that endangered her life”
	Reluctance to Disclose Previous Treatments (*n* = 2)	“She denied any trauma and being injected with another dermal filler in the forehead during history taking,” “reluctant to disclose previous treatment”
	Engagement in Illegal Surgeries (*n* = 1)	“it may lead to illegal surgeries performed by non-professional medical specialists”
Coping mechanisms for negative emotions	Relief from Distress (*n* = 3)	“In order to relieve distress,” “an action that procures temporary relief and response”
	Enhancing Low Self-Esteem (*n* = 4)	“Temporarily enhance her poor self-esteem,” “low self-esteem, perfectionism, and anxiety, may also contribute to the development of addictive behaviours”
	Reducing Anxiety (*n* = 3)	“Undertaken to prevent or reduce anxiety”
	Resolving Interpersonal Conflicts (*n* = 2)	“To escape from family tensions,” “resolving intra- and interpersonal conflicts”
Related psychological problems	Coexistence with Mental Disorders (*n* = 7)	“Several of these patients have under-recognized or untreated psychiatric disorders,” “closely associated with body dysmorphic disorder (BDD)”
	Perfectionism (*n* = 5)	“The pursuit of perfection,” “never seen as being good enough”
	Pathological Lying (*n* = 2)	“She used all her available credit cards, stole money from her parents and her friends and posed naked, all to alleviate her suffering and attempt to satisfy her addiction”
	Psychological dependence (*n* = 2)	“These changes in psychological state may support a potential neurobiological reward cycle activation after interventional aesthetic enhancement,” “withdrawal symptoms similar to those of drug addicts if they do not have at least two surgeries a year.”
Risk factors	Undergoing cosmetic procedures (*n* = 3)	“One risk of cosmetic surgery is that it can lead to cosmetic surgery addiction,” “series of cosmetic surgery may lead to cosmetic surgery addiction”
Future needs	Need for proper screening (*n* = 2)	“Proper screening and evaluation of these patients could save money and resources,” “emphasized the importance of histopathology for making an accurate diagnosis”
	Need for Comprehensive Management Strategies (* n* = 2)	“Collaborative efforts between dental professionals and mental health practitioners can facilitate a holistic approach”
	Need for Preventive Measures (*n* = 1)	“The design of therapeutic programs to prevent cosmetic surgery addiction and education programs to improve body appreciation”

Another defining characteristic, as identified in the reviewed studies, is a preoccupation with undergoing cosmetic procedures, which includes “obsessive” (*n* = 6) and “compulsive” (*n* = 4) tendencies. The term “obsessive” frequently appeared in relation to appearance, such as the “growing obsession with appearance” [38], while “compulsive” often described the approach to these procedures, as seen in the “compulsive need for surgical interventions” [35]. These characteristics often overlap; for example, one study described addiction to a teeth whitening procedures as “people who engage in excessive and compulsive behaviors” [39].

The reviewed studies also mentioned the characteristic of hazardous use of cosmetic procedures, which includes the “denial of potential risks” (*n* = 5). In these cases, it was mentioned that individuals may push aside potential risks and complications to continue pursuing cosmetic procedures [40]. Additionally, some of the reviewed studies mention “increasing adverse outcomes” (*n* = 2), such as the increasing risk of adverse reactions from repeated, excessive use [10], alongside a “reluctance to disclose previous treatment” (*n* = 2), where individuals may deny past procedures or trauma [11], and “engagement in illegal surgeries” (*n* = 1), often performed by non-professional medical specialists [41].

In the reviewed studies, addiction to cosmetic procedures is also associated with various coping mechanisms for negative emotions. For instance, some studies discuss “seeking relief from distress” (*n* = 3), where individuals engage in cosmetic procedures as a means of alleviating psychological distress. Similarly, “enhancing low self-esteem” (*n* = 4) is also mentioned in the reviewed studies, as individuals may seek cosmetic procedures to temporarily improve their self-image [37]. Other studies in the reviewed literature highlight the role of cosmetic procedures in “reducing anxiety” (*n* = 3), where cosmetic procedures are undertaken to prevent or reduce anxiety [42], and to “resolve interpersonal conflicts” (*n* = 2), where individuals may use these procedures as a means of escaping family tensions [37].

Various psychological problems are related to addiction to cosmetic procedures in the reviewed studies. For example, the “coexistence with mental disorders” (*n* = 7) was often mentioned. Several studies specifically recognized the association with body dysmorphic disorder (BDD). However, it is also mentioned in the reviewed studies that cosmetic procedures often fail to improve the symptoms of BDD, as the underlying psychological issues remain unaddressed [35]. Perfectionism (*n* = 5) was another psychological issue linked to cosmetic procedure addiction, where the “quest for physical perfection” [10] can drive individuals to undergo multiple procedures, leaving them dissatisfied over results [37, 43]. Pathological lying (*n* = 2) is another psychological problem that is related to addiction to cosmetic procedures in the reviewed studies. Finally, a psychological problem that is discussed in the reviewed literature is the “psychological dependence” (*n* = 2) on cosmetic procedures, denoting reward effects and withdrawal symptoms often associated with behavioral addictions [44]. For instance, one study discusses a potential neurobiological reward cycle activation after interventional esthetic enhancement [45]. This psychological dependence also relates to withdrawal symptoms, as another study discusses that individuals may exhibit withdrawal symptoms similar to those of drug addicts, if they do not undergo at least two surgeries per year [37]. Importantly, however, these studies do not explain what these withdrawal symptoms entail for cosmetic procedure recipients.

The reviewed literature also identifies “undergoing cosmetic procedures” as a potential risk factor for developing an addiction to these procedures (*n* = 3). For instance, one study suggests that undergoing a series of cosmetic procedures may increase the likelihood of developing an addiction to cosmetic procedures [41]. Additionally, another study highlights that the positive outcomes people experience from cosmetic procedures, which promote their appearance-enhancing behaviors, could contribute to the risk of addiction to cosmetic procedures [35].

In addition to these key characteristics of cosmetic procedures, the reviewed literature highlights future needs to address addiction to cosmetic procedures. As mentioned in the reviewed studies, there is a recognized “need for proper screening” (*n* = 2) to identify under-recognized or untreated psychiatric disorders, which could save resources [11]. Furthermore, a “need for comprehensive management strategies” (*n* = 2) is emphasized, including the integration of mental health aspects in cosmetic care. In one of the reviewed studies, a collaborative approach between professionals and mental health practitioners has been suggested to ensure a holistic treatment strategy [39]. Lastly, the “need for preventive measures” (*n* = 1) is acknowledged in one of the reviewed studies, with recommendations for therapeutic programs to prevent addiction to cosmetic procedures and educational initiatives to improve body appreciation [41].

## Discussion

While cosmetic procedures continue to rise in popularity, there is a growing concern about the potential for addiction to cosmetic procedures. This review represents a critical step in examining the concept of addiction to cosmetic procedures. Our first aim was to compile and explore articles defining addiction to cosmetic procedures. Despite an extensive and thorough search across various search engines, our scoping review yielded a limited number of studies that specifically describe addiction to cosmetic procedures. This scarcity highlights the fact that the concept of addiction to cosmetic procedures remains an underexplored area in the literature. An additional issue relates to the various terminology to describe addictive tendencies associated with cosmetic procedures, with some studies referring to individuals as “surgery junkies” [46] or employing procedure-specific terms like “filler-addicted” [10]. The lack of a consistent definition makes it challenging to standardize the understanding of addiction to cosmetic procedures.

To strengthen the specificity of this scoping review, we used a directed content analysis, which enables the application of existing theories or frameworks while also enabling new evidence to emerge through open coding [33, 34]. Specifically, existing literature on cosmetic procedures [29, 30] and behavioral addictions [27, 31, 32] was used to guide our directed content analysis. This approach allowed us to identify key characteristics of cosmetic procedures that align with those seen in behavioral addictions [24], while also identifying additional characteristics highlighted in the reviewed literature.

Based on our analysis, we found that the most frequently mentioned characteristic is the tendency to undergo “multiple and repeated procedures” that are “excessive and out of proportion,” which often involves visits to several cosmetic practitioners [11] and a persistent obsession with altering additional body parts [37]. This behavior aligns with the criteria of behavioral addictions, specifically high(er) involvement over an extended period.

Other characteristics that align with behavioral addictions were also observed. The reviewed literature highlights obsessive and compulsive behaviors related to cosmetic procedures, reflecting a preoccupation with appearence [35, 38]. Furthermore, hazardous use patterns were described, with individuals continuing to seek cosmetic procedures despite potential risks, including engaging in illegal surgeries [41]. Psychological dependence on cosmetic procedures was also noted, with one study specifically mentioning withdrawal symptoms similar to those experienced by drug addicts if individuals do not undergo at least two surgeries per year [37]. These behaviors are aligning with behavioral addictions, suggesting that addiction to cosmetic procedures shares significant similarities with (other) behavioral addictions.

While behavioral addictions are considered under the “Substance-Related and Addictive Disorders” section of the DSM-5, gambling disorder remains the only formally recognized behavioral addiction. This limited recognition reflects the lack of data on other behavioral addictions, such as gaming and shopping [21, 24]. Although addiction to cosmetic procedures is not yet formally recognized, our review suggests that the “excessive” and “compulsive” use of cosmetic procedures shares many similarities with recognized behavioral addictions. Based on this review, it may be desirable to further conceptualize and investigate whether “addiction” to cosmetic procedures could and/or should be recognized as a behavioral addiction [24, 47]. As this scoping review is an initial step in exploring this possibility, future research should examine whether addiction to cosmetic procedures can be defined as a behavioral addiction and assessed using criteria similar to those for gambling disorder [24]. Additionally, it is crucial to consider what the implications of such a conceptualization would be.

Several key issues must be addressed in conceptualizing addiction to cosmetic procedures if further research is pursued. A critical question that remains unanswered is what constitutes “multiple” or “excessive” cosmetic procedures. While many studies in the reviewed literature discuss the excessive use of cosmetic procedures, none clarifies the threshold at which the frequency of these procedures becomes disproportionate and qualifies as an addiction. Additionally, it remains unclear whether this threshold varies depending on the procedure type and/or the scope and scale of procedures. For example, a distinction may be needed between revision surgeries to correct postoperative defects versus multiple unrelated procedures. Addressing this gap is essential for advancing our understanding and for developing standardized diagnostic criteria for addiction to cosmetic procedures. Additionally, the involvement of medically qualified personnel conducting these cosmetic procedures is crucial to identify when the quantity of procedures indicates addiction. Therefore, future research should aim to determine whether, and if so when, the quantity of cosmetic procedures indicates addiction, particularly across different types of procedures.

The lack of a standardized definition and diagnostic criteria for addiction to cosmetic procedures presents significant challenges. It complicates the identification of individuals at risk and hampers the development of effective screening and preventive measures. While some studies did discuss the need for proper screening [11] and preventive strategies [41], none of the studies provided specific guidelines or interventions. Future research should focus on establishing clear assessment criteria, which would enable the development of targeted screening tools and effective prevention strategies that are sensitive to the varying types of cosmetic procedures available.

In addition, if threshold criteria are established for conceptualizing cosmetic procedure addiction and this type of addiction is more widely accepted, understanding the precursors to addiction to cosmetic procedures is also crucial. Some of the reviewed studies identify factors such as perfectionism, anxiety and low self-esteem as potential precursors to addiction [39]. These factors align with those commonly seen in behavioral addictions, which often include psychopathologies such as social anxiety and perfectionism [23, 48]. Individuals with these psychological vulnerabilities might be at higher risk of developing addiction to cosmetic procedures, potentially driven by unrealistic expectations and dissatisfaction with the results. These may be further exacerbated by social media, as social media significantly influences beauty standards and increases demand for esthetic procedures [49]. Recognizing and further investigating these precursors is important for developing targeted interventions and preventive measures for addiction to cosmetic procedures.

Finally, the reviewed studies identified a critical risk factor for addiction to cosmetic procedures—namely undergoing cosmetic procedures. Although some studies suggest that undergoing a series of cosmetic procedures may increase the likelihood of developing an addiction, further research is needed to fully understand this relationship. This understanding is crucial for developing effective prevention strategies, as it can help identify individuals at higher risk for addiction to cosmetic procedures.

This review provides a necessary step toward conceptualizing addiction to cosmetic procedures. However, there are some limitations to our scoping review. Firstly, relevant studies may have been missed due to the specific keywords used in our search strategy. Secondly, we only included studies published in English, potentially overlooking research available in other languages. Additionally, although predefined inclusion and exclusion criteria were applied to guide the selection process, the review was conducted by two reviewers, which may have introduced potential bias. Disagreements between the reviewers were resolved through discussion, which should be considered a potential limitation. Despite these limitations, our review encompassed a broad range of study types, including cross-sectional studies and case reports, providing a comprehensive overview of the existing literature on addiction to cosmetic procedures.

## Conclusion

In conclusion, despite extensive efforts to compile research from various sources, the existing literature on addiction to cosmetic procedures is limited. The studies that do exist employed a variety of terms for addiction to cosmetic procedures. However, they consistently indicate the presence of addictive tendencies in the context of cosmetic procedures, such as increased use of (“excessive”) procedures, preoccupation with cosmetic procedures, hazardous use and psychological dependence on undergoing procedures. These tendencies align with established characteristics of behavioral addictions. Our review represents a critical step in conceptualizing addiction to cosmetic procedures. Further research is needed to explore whether addiction to cosmetic procedures could and/or should be recognized as a behavioral addiction and what the implications of this would be for the field.

## Supplementary Information

Below is the link to the electronic supplementary material.

PDF 154 kb)

## Footnotes

1 We introduce the term “addiction” to cosmetic procedures here. However, this term is not yet formally defined in the academic literature. For the purpose of this paper, we will use the term addiction to cosmetic procedures as a working concept. This means that we acknowledge that it refers to a newly emerging concept rather than an established and formal term.
