# Supplementary material for: Addiction to Cosmetic Procedures: A Scoping Literature Review
Source: Aesthetic Plast Surg. 2025 Aug 12;49(24):6923–31. doi: 10.1007/s00266-025-05153-8 (PMC12855224; doi:10.1007/s00266-025-05153-8)
Supplement: Supplementary file 1 — PDF 154 kb) [file 266_2025_5153_MOESM1_ESM.pdf]

Supplemental Table: Definitions of psychological terms

Term	Definition
Addiction	A process whereby a behavior, that can function both to produce pleasure and to provide relief from internal discomfort, is employed in a pattern characterized by (1) recurrent failure to control the behavior(powerlessness) and (2) continuation of the behavior despite significant negative consequences (un-manageability)[1].
Behavioral addiction	Instead of being addicted to a substance, a person is addicted to the behavior or the feeling experienced by acting out the behavior[2].
DSM	DSM is the standard classification of mental disorders used for clinical, research, policy, and reimbursement purposes in the United States and elsewhere[3].
Obsession	An idea or thought that continually preoccupies or intrudes on a person's mind[4].
Preoccupation	Stressor-related factual thinking, which is time-consuming and associated with negative emotions[5].
Psychological dependence	A compulsion to use or act to produce pleasure or to avoid discomfort, despite negative consequences[6].
Social anxiety	An intense fear of social situations in which the person may be scrutinized by others[7].
Tolerance	The need to increase the intensity or frequency of the behavior in order to achieve the desired effect or diminished effect with continued behavior of the same intensity[1].

References

1. Goodman A. Addiction: definition and implications. *British journal of addiction*. 1990;85(11):1403-1408.
2. Alavi SS, Ferdosi M, Jannatifard F, Eslami M, Alaghemandan H, Setare M. Behavioral addiction versus substance addiction: Correspondence of psychiatric and psychological views. *International journal of preventive medicine*. 2012;3(4):290.
3. Hasin DS, O'brien CP, Auriacombe M, et al. DSM-5 criteria for substance use disorders: recommendations and rationale. *American Journal of Psychiatry*. 2013;170(8):834-851. doi:10.1176/appi.ajp.2013.12060782
4. Salkovskis PM. Obsessions and compulsions. *Cognitive therapy in clinical practice*. Routledge; 2003:39-54.
5. Eberle DJ, Maercker A. Preoccupation as psychopathological process and symptom in adjustment disorder: A scoping review. *Clinical Psychology & Psychotherapy*. 2022;29(2):455-468.
6. Johnson KR, Fuchs E, Horvath KJ, Scal P. Distressed and looking for help: Internet intervention support for arthritis self-management. *J Adolesc Health*. Jun 2015;56(6):666-71. doi:10.1016/j.jadohealth.2015.02.019
7. Leichsenring F, Leweke F. Social anxiety disorder. *New England Journal of Medicine*. 2017;376(23):2255-2264.
